# Breast cancer stem cells in HER2-negative breast cancer cells contribute to HER2-mediated radioresistance and molecular subtype conversion: clinical implications for serum HER2 in recurrent HER2-negative breast cancer

**DOI:** 10.18632/oncotarget.23528

**Published:** 2017-12-20

**Authors:** Yun Gyoung Kim, Yi Na Yoon, Hyang Suk Choi, Ji-Hyun Kim, Hyesil Seol, Jin Kyung Lee, Min-Ki Seong, In Chul Park, Kwang Il Kim, Hyun-Ah Kim, Jae-Sung Kim, Woo Chul Noh

**Affiliations:** ^1^ Department of Surgery, Korea Cancer Center Hospital, Korea Institute of Radiological and Medical Sciences, Seoul, Korea; ^2^ Department of Surgery, Bundang Jesaeng General Hospital, Seongnam, Korea; ^3^ Division of Radiation Cancer Research, Korea Institute of Radiological and Medical Sciences, Seoul, Korea; ^4^ RI-Convergence Research, Korea Institute of Radiological and Medical Sciences, Seoul, Korea; ^5^ Radiological and Medico-Oncological Sciences, University of Science and Technology, Seoul, Korea; ^6^ Department of Pathology, Korea Cancer Center Hospital, Korea Institute of Radiological and Medical Sciences, Seoul, Korea; ^7^ Department of Laboratory Medicine, Korea Cancer Center Hospital, Korea Institute of Radiological and Medical Sciences, Seoul, Korea

**Keywords:** breast cancer, HER2-negative breast cancer, breast cancer stem cells, serum HER2, radioresistance

## Abstract

Although it has been proposed that the beneficial effect of HER2-targeted therapy in HER2-negative breast cancer is associated with the molecular subtype conversion, the underlying mechanism and the clinical biomarkers are unclear. Our study showed that breast cancer stem cells (BCSCs) mediated HER2 subtype conversion and radioresistance in HER2-negative breast cancer cells and evaluated serum HER2 as a clinical biomarker for HER2 subtype conversion. We found that the CD44^+^/CD24^–/low^ BCSCs from HER2-negative breast cancer MCF7 cells overexpressed HER2 and EGFR and showed the radioresistant phenotype. In addition, we showed that trastuzumab treatment sensitized the radioresistant phenotype of the CD44^+^/CD24^–/low^ cells with decreased levels of HER2 and EGFR, which suggested that HER2-targeted therapy in HER2-negative breast cancer could be useful for targeting BCSCs that overexpress HER2/EGFR. Importantly, our clinical data showed that serial serum HER2 measurement synchronously reflected the disease relapse and the change in tumor burden in some patients who were initially diagnosed as HER2-negative breast cancer, which indicated that serum HER2 could be a clinical biomarker for the evaluation of HER2 subtype conversion in patients with recurrent HER2-negative breast cancer. Therefore, our data have provided *in vitro* and *in vivo* evidence for the molecular subtype conversion of HER2-negative breast cancer.

## INTRODUCTION

Approximately 10–30 % of breast cancers show epidermal growth factor receptor 2 (HER2) amplification, which is associated with a high risk of relapse and poor treatment outcomes [1–3]. HER2-targeted therapy, such as trastuzumab, improves progression-free survival and overall survival in HER2-positive breast cancers [4]. Remarkably, recent retrospective studies have suggested that HER2-targeted therapy may also benefit patients with HER2-negative breast cancer [5, 6]. The HER2 status of metastatic lesions is not always concordant with that of the primary tumor. It was reported the HER2 status of the primary and metastatic lesions was concordant in only 66% of patients and that the rate of molecular subtype conversion from a primary HER2-negative tumor to HER2-positive metastatic breast cancer was 9.7% [7, 8], which suggested that the molecular subtype conversion of the primary breast cancer occurred during disease progression. This discordance of HER2 status between the primary tumor and metastatic lesion could deprive the patient of the opportunity of HER2-targeted therapy and lead to poorer survival outcomes. Unfortunately, the repeated biopsy of metastatic lesions required to evaluate HER2 status is difficult in a clinical setting. Therefore, it is necessary to understand the mechanism and biomarkers of HER2 subtype conversion.

Recently, several studies have suggested that breast cancer stem cells (BCSCs) could be involved in HER2 subtype conversion [9, 10]. It was reported that luminal BCSCs that do not display *HER2* gene amplification overexpressed HER2 and that trastuzumab treatment reduced luminal BCSCs [9]. In addition, other studies showed that irradiation induced HER2 expression in HER2-negative breast cancer cells and that the HER2 induction was associated with HER2^+^/CD44^+^/CD24^–/low^ BCSCs and radioresistance [10], which suggested that HER2 overexpression in HER2-negative breast cancer was associated with BCSCs and radioresistance. However, it is still unclear whether luminal BCSCs overexpress HER2 or another receptor family such as EGFR, as the hetero-dimerization of HER2 and EGFR is critical for the activation of the downstream pathway of HER2 [11]; if so, HER2-targeted therapy could eradicate luminal breast cancer stem cells in response to therapeutic treatments such as irradiation. In addition, it is important to develop clinical methods for the evaluation of the HER2 subtype conversion from the primary HER2-negative tumor during disease progression.

The extracellular domain of the HER2 protein may be shed into the serum and the clinical use of this has been investigated [2, 3, 12–14]. Serum HER2 testing has been approved by the Food and Drug Administration since 2000. Several reports have indicated that increased serum HER2 level is associated with a poor prognosis and acts as a predictive factor for HER2-targeted treatment resistance in HER2-positive tumors [14, 15]. In addition, our previous study indicated that serial serum HER2 measurement during the follow-up periods after curative surgery was a useful method to detect the disease recurrence in HER2-positive breast cancer [2]. However, the clinical value of serum HER2 levels in HER2-negative breast cancer is not well understood. Therefore, this study investigated the role of BCSCs in HER2 subtype conversion, the radioresistance of HER2-negative breast cancer cells, and analyzed whether serum HER2 could be a clinical biomarker for the evaluation of HER2 subtype conversion from advanced HER2-negative breast cancer.

## RESULTS

### HER2 and EGFR are overexpressed in CD44^high^/CD24^low^ cells enriched from HER2-negative breast cancer cells

**Figure 1:**
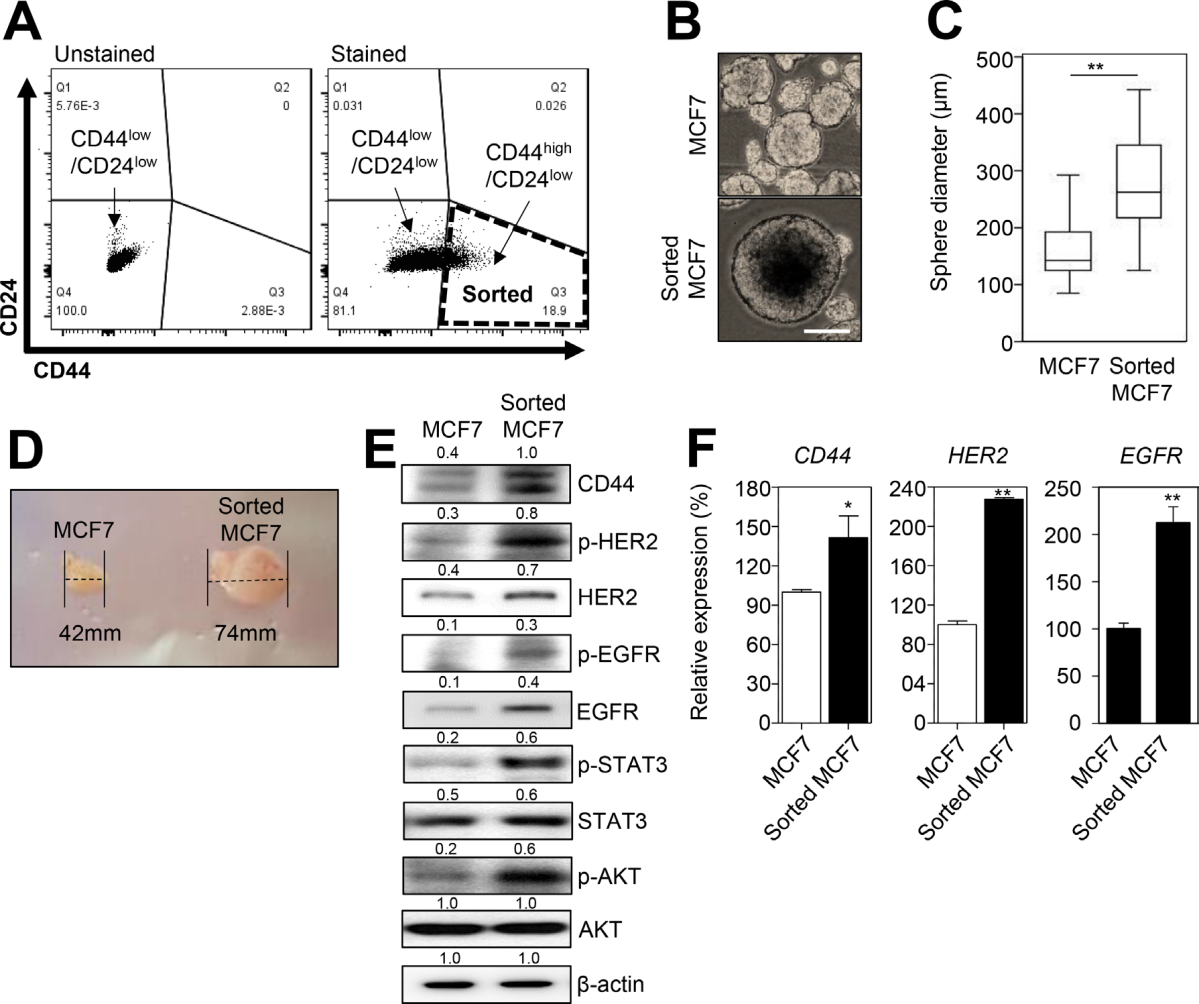
CD44^high^/CD24^low^ cells from HER2-negative breast cancer cells overexpress HER2 and EGFR (**A**) The flow cytometric analysis of MCF7 cells labeled without or with CD44-FITC and CD24-PE antibodies. The total MCF7 cells were divided into two subpopulations: CD44^high^/CD24^low^ and CD44^low^/CD24^low^. (B and C) The sphere forming assay was performed with MCF7 and sorted MCF7 (CD44^high^/CD24^low^) cells. 10^4^ cells were seeded in ultra-low adhesion plates and incubated for 7 days. Representative images of the sphere forming assay in MCF7 and sorted MCF7 cells (**B**). Scale bars, 100 μm. The sphere forming capacity was measured by the sphere diameter (μm) (**C**). (**D**) Representative images of the tumor size from xenograft assay by using MCF7 or sorted MCF7 cells. (**E**) MCF7 and sorted MCF7 cells were analyzed by immunoblotting with the indicated antibodies. The numbers indicate the quantification of the indicated protein expression. (**F**) The expression of CD44, HER2, and EGFR were measured by qRT-PCR. β-Actin (E) or GAPDH (F) was used as a loading control. The data represent typical results and were presented as the mean ± standard deviation of three independent experiments; *p* < 0.01 (^**^) and *p* < 0.05 (^*^).

To investigate whether HER2 was overexpressed in BCSCs from HER2-negative breast cancer cells, we first sorted the CD44^high^/CD24^low^, a marker for BCSCs [16], population from HER2-negative breast cancer MCF7 cells (Figure 1A). The mammosphere formation assay indicated that the CD44^high^/CD24^low^ MCF7 cells have a stem-like phenotype (Figure 1B and 1C). In addition, we found that the CD44^+^/CD24^low^ MCF7 cells were able to form tumors in BALB/c-nude mice (Figure 1D), which confirmed that the CD44^high^/CD24^low^ enriched cells were BCSCs. As the hetero-dimerization of HER2 and EGFR drives breast cancer progression and cancer stemness [11], we then observed the expression of CD44, HER2, and EGFR. We found that HER2, EGFR, and CD44 were overexpressed and activated in the CD44^high^/CD24^low^ MCF7 cells, as determined by western blot and qRT-PCR analysis (Figure 1E and 1F). Therefore, our data suggested that HER2 and EGFR were overexpressed in BCSCs of HER2-negative breast cancer cells.

### CD44^high^/CD24^low^ cells in HER2-negative breast cancer cells are associated with radiation-resistant phenotype

**Figure 2:**
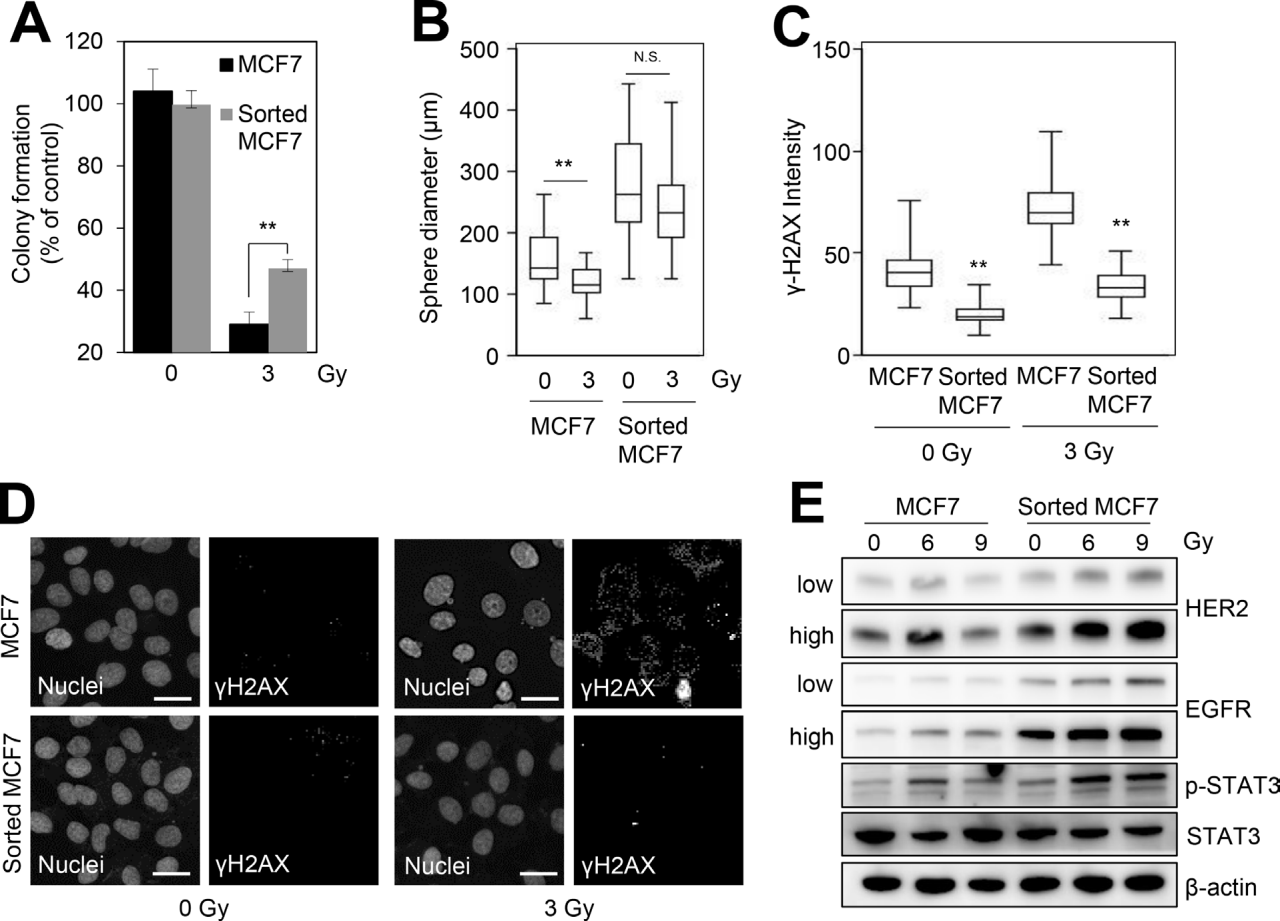
CD44^high^/CD24^low^ cells from HER2-negative breast cancer cells are associated with radioresistance MCF7 and sorted MCF7 cells were irradiated with 3 Gy and then we performed a colony formation assay (**A**), sphere forming assay (**B**), or immunofluorescence analysis (**C**). (A) The number of colonies was measured and presented as the values relative to that of the control. (B) The sphere forming capacity was measured by the sphere diameter (μm). (C) The fluorescence intensity of γ-H2AX was quantified by using an IN Cell Analyzer 2200. (**D**) Representative images of γ-H2AX foci in the control and the irradiated cells. Scale bars, 20 μm. (**E**) MCF7 and sorted MCF7 cells were treated with the indicated doses of irradiation for 24 h and then analyzed by immunoblotting with the indicated antibodies. “Low” or “high” indicate low or high exposure of the bands, respectively. β-Actin was used as the loading control. The data represent the typical results and are presented as the mean ± standard deviation of three independent experiments; *p* < 0.01 (^**^). N.S.: not significant.

As BCSCs are known to be associated with radioresistance [10], we further investigated the role of the CD44^high^/CD24^low^ MCF7 cells enriched from HER2-negative breast cancer cells in radioresistance. The colony forming assay indicated that the CD44^high^/CD24^low^ MCF7 cells were more resistant to irradiation in comparison with control MCF7 cells (Figure 2A). In addition, the sphere-forming capacity in response to irradiation was higher in CD44^high^/CD24^low^ MCF7 cells compared with that in the control MCF7 cells (Figure 2B). γ-H2AX analysis also indicated that CD44^high^/CD24^low^ MCF7 cells had a superior capacity to repair IR-induced DNA damage compared with the control MCF7 cells (Figure 2C and 2D). In addition, we found that irradiation significantly induced HER2/EGFR expression and the activation of STAT3, which is a downstream effector of HER2 signaling [10, 17], in CD44^high^/CD24^low^ MCF7 cells compared with the control MCF7 cells (Figure 2E). Thus, these data suggested that the BCSCs of HER2-negative breast cancer cells yielded the radioresistant phenotype with increased HER2 and EGFR.

### Trastuzumab treatment sensitizes the radioresistant phenotype of CD44^high^/CD24^low^ cells enriched from HER2-negative breast cancer cells

**Figure 3:**
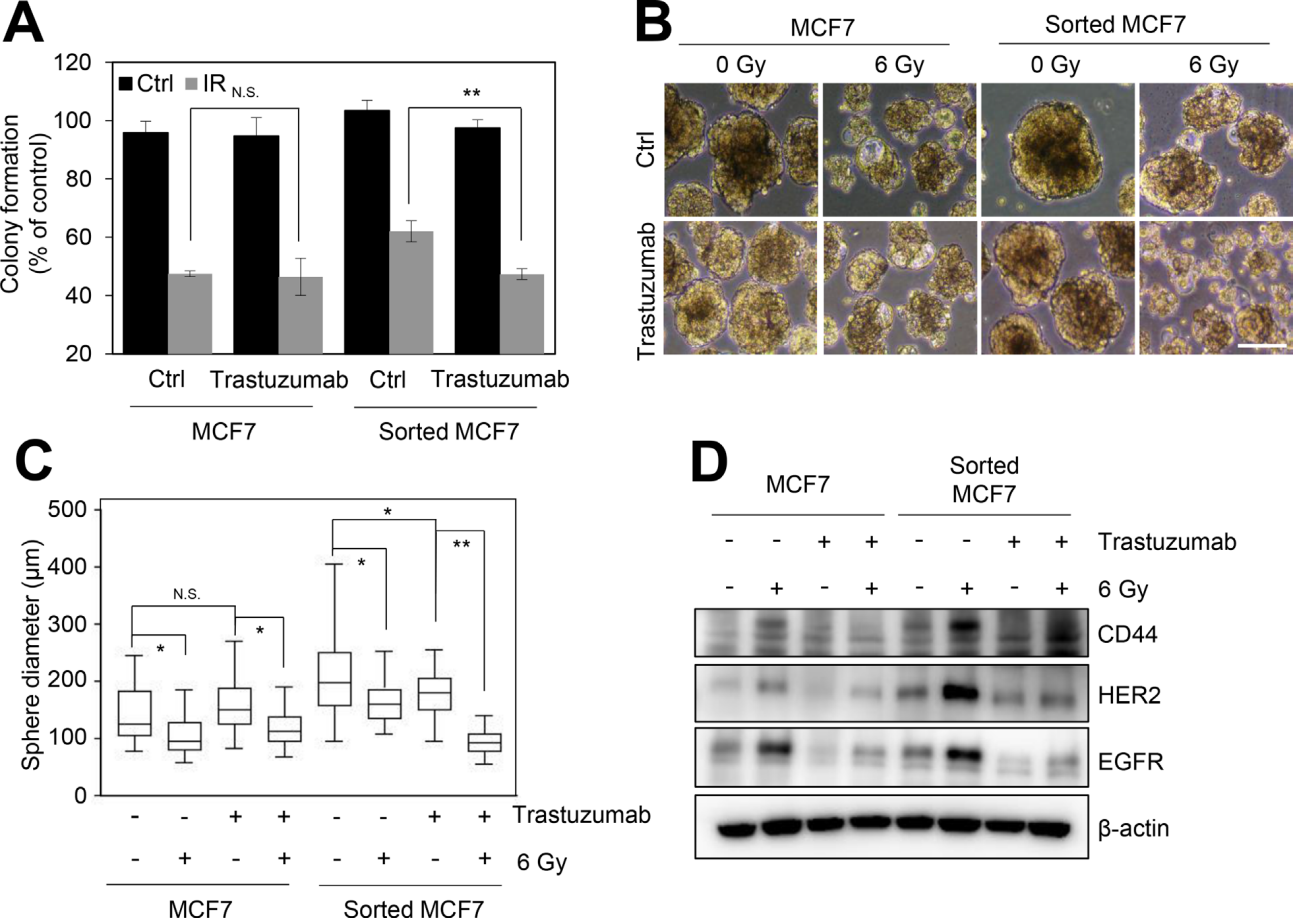
Trastuzumab treatment reduces the radioresistance of the CD44^high^/CD24^low^ cells from HER2-negative breast cancer cells MCF7 and sorted MCF7 cells were treated without or with 10 μg/mL trastuzumab for 16 h, and further treated with 3 Gy irradiation. (**A**) The colony formation assay was performed. The number of colonies was measured and presented as relative values to the control. (**B**) The sphere forming assay was performed. Scale bars, 200 μm. Representative images of sphere forming assay in the MCF7 and sorted MCF7 cells (B). The sphere forming capacity was measured from the sphere diameter (μm) (**C**). (**D**) The cells were analyzed by immunoblotting with the indicated antibodies. β-Actin was used as a loading control. The data represent typical results and are presented as the mean ± standard deviation of three independent experiments; *p* < 0.01 (^**^) and *p* < 0.05 (^*^). N.S.: not significant.

In response to irradiation, CD44^high^/CD24^low^ MCF7 cells significantly increased the expression of HER2 and EGFR (Figure 2B); therefore, we examined whether HER2-targeted therapy could sensitize the radioresistant phenotype of CD44^high^/CD24^low^ MCF7 cells. The colony forming assay indicated that the combination treatment of irradiation and trastuzumab sensitized CD44^high^/CD24^low^ MCF7 cells, but not control MCF7 cells (Figure 3A). The sphere forming capacity of CD44^high^/CD24^low^ MCF7 cells was also significantly reduced by the combination treatment of radiation and trastuzumab (Figure 3B and 3C). In addition, we found that the combination treatment of radiation and trastuzumab reduced the expression of CD44, HER2, and EGFR (Figure 3D). Therefore, our data suggested that HER2-targeted therapy using trastuzumab was able to overcome the radioresistant phenotype of BCSCs in HER2-negative breast cancer cells, which provided an indication that the clinical benefit of HER2-targeted therapy in patients with recurrent HER2-negative breast cancer might be associated with BCSCs.

### Serum HER2 is a surrogate marker for HER2 subtype conversion from primary HER2-negative tumors

**Figure 4:**
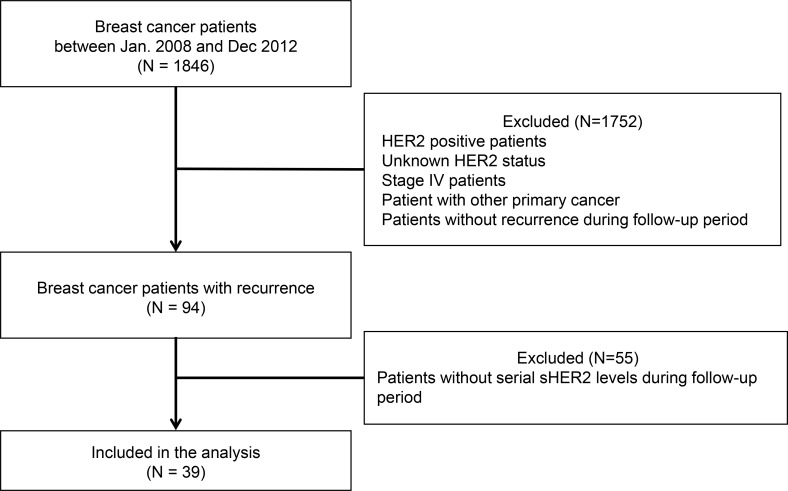
Flow diagram for the patient selection process The study patients were selected from 1,846 patients who underwent curative surgery at the Korea Cancer Center Hospital between January 2008 and December 2012. The detailed process is described in Material and Methods section.

**Figure 5:**
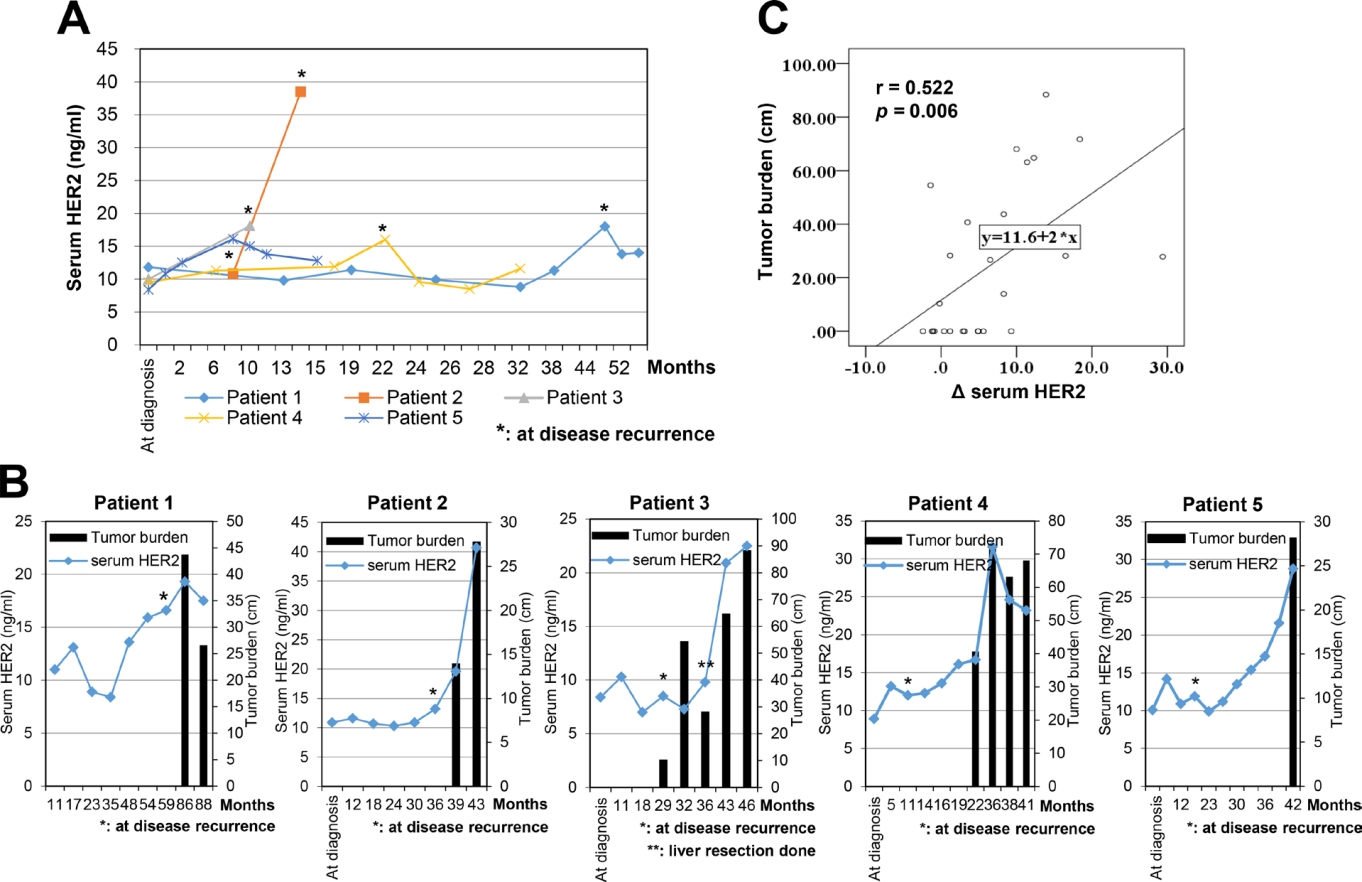
Serum HER2 levels in patients with HER2-negative breast cancer (**A**) The correlation of serum HER2 level and tumor burden in metastatic disease. Tumor burden was defined by the longitudinal diameter of the measurable lesions (cm). (**B**) Patient 1: A 41-year-old woman was diagnosed with multiple lung and lymph node metastasis at 59 months after curative surgery. Patient 2: A 53-year-old woman was detected to have multiple bone and lung metastasis at 36 months after a total mastectomy. After 3 months, bone and lung lesions had progressed. Patient 3: A 52-year-old woman presented with liver metastasis at 21 months after operation. Patient 4; A 57-year-old woman was diagnosed with lung and bone metastasis at 11 months after surgery. Patient 5; A 50-year-old woman was discovered to have bone metastases 17 months after a total mastectomy. (**C**) The relationship between serum HER2 levels and tumor burden.

As our previous data suggested that serum HER2 levels were observed in patients with relapsed HER2-negative breast cancer [12], we next examined whether serum HER2 could act as a clinical biomarker for HER2 subtype conversion from the primary HER2-negative tumor during metastasis or disease progression. We initially analyzed 1,846 patients with invasive ductal carcinoma or invasive lobular carcinoma, and then excluded HER2-positive patients, patients with unknown HER2 status, and patients with other primary cancers. Patients without the results of serial serum HER2 levels were also excluded from this study, which yielded a total of 39 patients (Figure 4). The basic characteristics of patients are shown in Table 1. Ten patients (25.6%) developed local or regional recurrence and distant metastases were found in 29 patients (74.4%) during the follow-up period. Although all patients had HER2-negative primary tumors, increased serum HER2 was observed at least once after the disease recurrence in 13 patients (33.3%). At the time of disease recurrence, increased serum HER2 level (Figure 5A) was observed in five patients (12.8%), all of whom had distant metastases in which the metastatic sites were lung, bone, or liver. The serum HER2 levels tended to decrease after the start of systemic treatment for metastatic disease. In eight patients (20.5%), serum HER2 level increased synchronously with disease progression. These patients had normal serum HER2 level at the time of initial diagnosis and initial recurrence, but the levels fluctuated simultaneously with the metastatic tumor burden in each patient. Among these patients, five patients who had a measurable tumor according to the Response Evaluation Criteria in Solid Tumors (RECIST) criteria, showed that serum HER2 level tended to increase with an increase in tumor burden (Figure 5B).

**Table 1:** Patients characteristics

	Patients (*N* = 39)	
	N	%
Age		
<50 years	15	38.5
≥50 years	24	61.5
T Stage		
T1	9	23.1
T2	27	69.2
T3	1	2.6
T4	2	5.1
N stage		
N0	8	20.5
N1	13	33.3
N2	11	28.2
N3	7	17.9
Unknown		
Histologic grade		
Grade I	2	5.1
Grade II	16	41.0
Grade III	18	46.2
Unknown	3	7.7
Hormone receptor		
ER+ and/or PR+	27	69.2
ER- and PR-	12	30.8
HER2 status		
Negative on IHC	17	43.6
1+ on IHC	17	43.6
2+ on IHC and negative on FISH	5	12.8

ER: estrogen receptor, PR: progesterone receptor, HER2: human epidermal growth factor receptor-2, IHC: immunohistochemistry, FISH: fluorescence *in situ* hybridization

Next, we evaluated the relationship between the change in serum HER2 level and tumor burden. The Δ serum HER2 level was defined as the difference between the average pre-recurrence serum HER2 level and each measured serum HER2 level after recurrence. The Δ serum HER2 level was positively associated with tumor burden, as measured synchronously by computed tomography (CT) and blood sampling for serum HER2 examination (Figure 5C, correlation coefficient = 0.522, *p* = 0.006). Therefore, our results suggested that serum HER2 could be a clinical biomarker for HER2 subtype conversion from the primary HER2-negative tumor during metastasis or disease progression, which provided clinical evidence that HER2-targeted therapy could be useful for patients with recurrent HER2-negative breast cancer who were diagnosed with a high level of serum HER2.

## DISCUSSION

In this study, we showed that CD44^+^/CD24^–/low^ BCSCs enriched from HER2-negative breast cancer cells displayed a radioresistant phenotype with HER2 and EGFR overexpression. In addition, we showed that HER2-targeted treatment using trastuzumab overcame the radioresistance of the CD44^+^/CD24^–/low^ BCSCs. Furthermore, our data indicated that serum HER2 could be a clinical biomarker for the evaluation of the HER2 subtype conversion from the primary HER2-negative tumor. Therefore, our data provide *in vitro* and *in vivo* evidence of HER2 subtype conversion from a HER2-negative tumor to a HER2-positive tumor, which support the use of HER2-targeted therapy for patients with recurrent HER2-negative breast cancer.

Recent retrospective studies revealed that HER2-targeted therapies were beneficial for patients with HER2-negative breast cancer [5, 6]. Although the underlying mechanisms for the clinical efficacy of adjuvant trastuzumab in HER2-negative breast cancer are still unclear, it has been proposed that cancer stem cells might be involved in the molecular subtype conversion from HER2-negative tumors to HER2-positive tumors [9, 10]. Ithimakin *et al.* reported that a high expression of HER2 was correlated with increased levels of aldehyde dehydrogenase activity in luminal breast cancer cells [9]. In addition, Duru *et al.* reported that HER2^+^/CD44^+^/CD24^–/low^ BCSCs from MCF7/C6 cells enhanced radioresistance and aggressiveness [10]. Similarly, our results showed that the CD44^+^/CD24^–/low^ BCSCs from HER2-negative breast cancer cells were associated with HER2 and EGFR overexpression and the radioresistant phenotype. Compared to *HER2* amplification in HER2-positive cancer cells, the increased levels of HER2 in the CD44^+^/CD24^–/low^ BCSCs were relatively low because these changes may be associated with altered epigenetic regulation [18, 19]. Recently, several studies indicated that BCSCs and other tumor cells, which may share genetic information, display distinct gene expression profiles due to epigenetic regulation, which could be induced by the tumor microenvironment [9, 19], suggesting that the relatively increased levels of HER2 and EGFR in the CD44^high^/CD24^low^ cells may be associated with epigenetic regulation. Furthermore, recent studies reported that the epigenetic modulation of cancer stem cells was tightly associated with cellular heterogeneity and plasticity in a variety of cancers, including breast cancer [20]. Therefore, it is likely that the survival of BCSCs from HER2-negative tumors during disease progression or therapeutic treatment contributes to HER2 subtype conversion from the primary HER2-negative tumor.

Previous work by our group, in addition to that of others, has shown that HER2 status was associated with radiotherapy resistance and that HER2 inhibition could enhance radiation sensitivity in HER2-positive breast cancer [17, 21, 22]; however, the trastuzumab-enhanced radiation sensitivity in HER2-negative breast cancer was considered a controversial topic [4]. Although it is accepted that HER2-targeted therapy does not respond to HER2-negative breast cancer cells [4], our data indicated that trastuzumab treatment sensitized the radioresistant phenotype of CD44^+^/CD24^–/low^ MCF7 cells. Similarly, Duru *et al.* reported that HER2 inhibition by siRNA enhanced the radiation sensitivity of HER2^+^/CD44^+^/CD24^–/low^ BCSCs from MCF7/C6 cells [10]. Therefore, our data provide the preclinical evidence that adjuvant HER2-targeted therapy could enhance the radiotherapy efficacy in patients with recurrent HER2-negative breast cancer with elevated serum HER2.

As HER2-targeted therapies could be useful for the subtype of converted patients among patients with HER2-negative breast cancer, it is critical to develop a method to identify patients with relapsed HER2-negative breast cancer who respond to HER2-targeted therapy. The measurement of serum HER2 has several clinical benefits, such as non-invasiveness, high sensitivity, specificity, and diagnostic accuracy in comparison with immunohistochemistry (IHC) and fluorescence *in situ* hybridization (FISH) analysis [3, 23]. Although the serum HER2 testing has failed to succeed as a standard clinical test to determine the eligibility of HER2-targeted therapies, many studies have demonstrated the correlation of serum HER2 levels with tissue HER2 status, disease stage, and disease-free survival [3, 14, 23, 24]. In addition, our previous study suggested that serum HER2 levels could be a useful real-time marker for tumor burden and recurrence in patients with HER2-positive breast cancer [12]. In this study, our data further indicated that serial serum HER2 measurement could synchronously reflect the disease relapse in HER2-negative breast cancer. In addition, the change in serum HER2 level was related to the change in tumor burden in these patients. Similarly, Felm *et al.* reported that increased serum HER2 level in primary HER2-negative breast cancer tissues were observed in 45% (12/53) of patients [25]. Therefore, it is likely that serum HER2 could be a clinical biomarker for HER2 subtype conversion from the primary HER2-negative tumor during metastasis or therapeutic treatment. In addition, our data provided a clinical rationale that patients with elevated serum HER2 should be considered for HER2-targeted therapy, even in HER2-negative advanced breast cancer.

In conclusion, our data suggested that BCSCs from HER2-negative breast cancer cells contributed to HER2-mediated radioresistance and HER2 subtype conversion from the primary HER2-negative tumor during disease progression, and that serum HER2 testing could be a clinical method to select the patients with HER2-negative advanced breast cancer that are suitable for HER2-targeted therapy. In addition, our work provides preclinical evidence to support the use of HER2-targeted therapies for patients with HER2-negative breast cancer.

## MATERIALS AND METHODS

### Cell lines and reagents

MCF7 cells were purchased from the American Type Culture Collection (Manassas, VA) and cultured in DMEM (Gibco-BRL, Rockville, MD) supplemented with 10% FBS (Corning, NY) and 1% penicillin/streptomycin at 37°C in a humidified 5% CO_2_ incubator. The cells were preserved and passaged for no longer than 2 months, in accordance with the ATCC protocol, with monthly tests for mycoplasma contamination conducted by DAPI staining. The cells were treated with trastuzumab (Roche, Basel, Switzerland) for 16 h before irradiation. The cells were irradiated by a ^137^Cs γ-ray source (Atomic Energy of Canada Ltd, Mississauga, Canada) at a dose rate of 3.81 Gy/min.

### BCSC sorting

MCF7 cells were washed once with PBS and harvested with trypsin-EDTA. The detached cells were washed with PBS supplemented with 5% fetal bovine serum, 1% penicillin/streptomycin, and 0.1% sodium azide (wash buffer), and then re-suspended in wash buffer. Fluorochrome-conjugated monoclonal antibodies, including fluorescein isothiocyanate (FITC)-anti-human CD44 (eBioscience Inc, San Diego, CA), phycoerythrin (PE)-anti-human CD24 (eBioscience), or their respective isotype controls, were added to the cell suspension at concentrations recommended by the manufacturer, and incubated at 4°C for 30 min in the dark. The labeled cells were washed in wash buffer and sorted into CD44^+^/CD24^–^ and CD44^+^/CD24^+^ subpopulations based on their surface markers by using a FACS Aria II (BD Biosciences, San Diego, CA). The cells were routinely sorted twice and then re-analyzed for purity.

### Quantitative RT-PCR

Quantitative RT-PCR was performed in accordance with a previously described protocol [26]. In brief, total RNA was isolated by Qiazol reagent (Qiagen, Valencia, CA) and reverse-transcribed using ImProm-II™ reverse transcription system (Promega, Madison, WI). Quantitative RT-PCR was performed in triplicate by using a Chromo 4 cycler (Bio-Rad, Richmond, CA) and SYBR Premix Ex Taq^™^ (Takara Bio, Shiga, Japan). The amplification signal from the target gene was normalized to that of GAPDH in the same reaction. The following PCR primers were employed for conventional PCR: CD44, 5′-CGGACACCATGGACAAGTTT-3′ (sense) and antisense 5′-GAAAGCCTTGCAGAGGTCAG-3′ (anti-sense); HER2, 5′-CATATGTCTCCCGCCTTCTG-3′ (sense) and 5′-CCCACACAGTCACACCATAAC-3′ (anti-sense); EGFR, 5′-CGTCGCTATCAAGGAATTAAG-3′ (sense) and 5′-TGGTGGGTATAGATTCTGTG-3′ (anti-sense); and glyceraldehyde 3-phosphate dehydrogenase (GAPDH), 5′-CATCTCTGCCCCCTCTGCTGA-3′ (sense) and 5′-GGATGACCTTGCCCACAGCCT-3′ (anti-sense).

### Colony forming assay

The colony forming assay was performed in accordance with a previously described procedure [26]. In brief, 1,000 cells were seeded in triplicate in 60 mm tissue culture dishes and treated with 10 μg/mL trastuzumab for 16 h. The cells were then exposed to the indicated radiation and the medium was changed to complete growth medium. After culture for approximately 14 days, the colonies were fixed with methanol and stained with trypan blue solution. The colonies containing more than 50 cells were counted as surviving colonies.

### Sphere forming assay

Cells (10,000 cells) were plated in ultra-low attachment plates (Corning, NY) in serum-free DMEM-F12 medium (Gibco Laboratories, Grand Island, NY) supplemented with 1:50 B-27 (Invitrogen, Carlsbad, CA), 20 ng/mL FGF (R&D Systems, Minneapolis, MN), and 20 ng/mL EGF (R&D Systems) and treated without or with 10 μg/mL trastuzumab. After 7 days, the spheres were fixed with methanol. The average sphere number and size were calculated by using ImageJ software and spheres with a diameter less than 50 mm were excluded from the analysis.

### Antibodies and western blot analysis

The following antibodies were used: mouse monoclonal antibodies against phospho(Tyr1068)-EGFR (Cell Signaling Technology Inc.), STAT3, and β-actin (Santa Cruz Biotechnology Inc., CA); rabbit monoclonal antibodies against CD44, phospho(S473)-Akt (Abcam, Cambridge, UK), EGFR, phospho(Thr705)-STAT3, and HER2 (Cell Signaling Technology Inc.); and rabbit polyclonal antibodies against Akt and phospho(Tyr1221/1222) -HER2 (Cell Signaling Technology Inc.).

Western blotting was performed as previously described [27]. In brief, the proteins were separated by SDS-polyacrylamide gel electrophoresis, transferred to a polyvinylidene fluoride membrane, and detected by using specific antibodies. The blots were developed by using HRP-conjugated secondary antibodies and enhanced chemiluminescence detection system (Amersham Life Science, Piscataway, NJ). The images of the bands were obtained by using an Amersham Imager 600 system (GE healthcare).

### γ-H2AX analysis

The immunofluorescence analysis was performed as previously described [27]. In brief, the cells were fixed with 4% paraformaldehyde, permeabilized, and blocked with 0.1% Triton X-100 and 5% fetal calf serum in PBS. The fixed cells were incubated with a primary antibody against γ-H2AX (Millipore, 05-636) and a secondary antibody against anti-mouse Alexa-488 (Molecular Probes, Eugene, OR). The images were obtained and analyzed by using an IN Cell Analyzer HCA Systems (IN Cell Analyzer 2200, GE Healthcare Life Sciences).

### Mouse xenograft

Female BALB/c-nude mice were purchased from Orient Bio (Seoul, South Korea). Four-week-old mice were transplanted with 5 × 10^6^ cells suspended in 50 μL PBS and mixed in a 1:1 ratio with Matrigel (Corning). The cancer cells were subcutaneously injected into the hind flank of the right rear leg of mice. The mice were administered 400 μg/kg water soluble-β-estradiol (Sigma) diluted in desterilized water by oral gavage every second day for 4 weeks. The mice were then sacrificed by CO_2_ inhalation and the xenograft tumors were dissected. The animal experiments were conducted under the guidelines approved by the institutional animal care and treatment committee of KIRAMS.

### Patients

The data of 1,846 patients with invasive ductal carcinoma or invasive lobular carcinoma who underwent curative surgery at the Korea Cancer Center Hospital between January 2008 and December 2012 were investigated in this study. HER2 status was determined by IHC or FISH. HER2 negativity was defined by an IHC rating of negative or 1+, or if the IHC rating was 2+, the FISH ratio was 1.8–2.2 [28]. The exclusion criteria were HER2 positive patients, patients with an unknown HER2 status, patients with other primary cancers, and patients without the results of serial serum HER2 level; a total of 39 patients were eligible. The pathologic stage was classified by the 7th American Joint Committee on cancer tumor node metastasis (TNM) stage. Locoregional recurrence or distant metastasis was confirmed by a biopsy of the metastatic site or by the following imaging methods: CT, ultrasonography (US), mammography, magnetic resonance image (MRI), bone scan, and positron emission tomography-computed tomography (PET-CT). This study was approved by Korea Cancer Center Hospital Institutional Review Board (K-1504-022-065).

### Serum HER2 measurement

Serum HER2 level was measured by the ADVIA^®^ Centaur immunoassay analyzer. This method measures the circulating levels in the blood of the extracellular domain of the HER2 by using two monoclonal antibodies. On the basis of a previous study conducted at our center, the cut-off level was 15.0 ng/mL. The detailed method to measure the serum HER2 level has been described previously [12].

### Measurement of tumor burden in patients with disease recurrence

The RECIST guideline version 1.1 was used for the evaluation of the relationship between the tumor burden and the serum HER2 level. The tumor burden was measured via CT, which was collected on the same day as blood sampling for the serum HER2 measurement. The tumor burden was assumed from the use of the sum of the longest diameters of all measurable lesions [29].

### Statistical analysis

The two-tailed Student’s *t*-test was performed to analyze the statistical differences between groups. The correlation between serum HER2 levels and the tumor burden was analyzed by using Spearman’s Rho correlation analysis. A value *p* < 0.05 was considered statistically significant. Statistical analyses were computed by Excel and XLSTAT software.

## Abbreviations

BCSCs: breast cancer stem cells
CT: computed tomography
EGFR: epidermal growth factor receptor
FISH: fluorescence *in situ* hybridization
HER2: epidermal growth factor receptor 2
IHC: immunohistochemistry
RECIST: response evaluation criteria in solid tumors
US: ultrasonography

## References

[1] Burstein HJ (2005). The distinctive nature of HER2-positive breast cancers. N Engl J Med.

[2] Ha JH, Seong MK, Kim EK, Lee JK, Seol H, Lee JY, Byeon J, Sohn YJ, Koh JS, Park IC, Noh WC, Kim HA (2014). Serial Serum HER2 Measurements for the Detection of Breast Cancer Recurrence in HER2-Positive Patients. Journal of Breast Cancer.

[3] Leyland-Jones B, Smith BR (2011). Serum HER2 testing in patients with HER2-positive breast cancer: the death knell tolls. Lancet Oncol.

[4] Arteaga CL, Sliwkowski MX, Osborne CK, Perez EA, Puglisi F, Gianni L (2011). Treatment of HER2-positive breast cancer: current status and future perspectives. Nat Rev Clin Oncol.

[5] Paik S, Kim C, Wolmark N (2008). HER2 status and benefit from adjuvant trastuzumab in breast cancer. N Engl J Med.

[6] Perez EA, Reinholz MM, Hillman DW, Tenner KS, Schroeder MJ, Davidson NE, Martino S, Sledge GW, Harris LN, Gralow JR, Dueck AC, Ketterling RP, Ingle JN (2010). HER2 and chromosome 17 effect on patient outcome in the N9831 adjuvant trastuzumab trial. J Clin Oncol.

[7] Regitnig P, Schippinger W, Lindbauer M, Samonigg H, Lax SF (2004). Change of HER-2/neu status in a subset of distant metastases from breast carcinomas. J Pathol.

[8] Lower EE, Glass E, Blau R, Harman S (2009). HER-2/neu expression in primary and metastatic breast cancer. Breast Cancer Res Treat.

[9] Ithimakin S, Day KC, Malik F, Zen Q, Dawsey SJ, Bersano-Begey TF, Quraishi AA, Ignatoski KW, Daignault S, Davis A, Hall CL, Palanisamy N, Heath AN (2013). HER2 drives luminal breast cancer stem cells in the absence of HER2 amplification: implications for efficacy of adjuvant trastuzumab. Cancer Res.

[10] Duru N, Fan M, Candas D, Menaa C, Liu HC, Nantajit D, Wen Y, Xiao K, Eldridge A, Chromy BA, Li S, Spitz DR, Lam KS (2012). HER2-associated radioresistance of breast cancer stem cells isolated from HER2-negative breast cancer cells. Clin Cancer Res.

[11] Moasser MM (2007). The oncogene HER2: its signaling and transforming functions and its role in human cancer pathogenesis. Oncogene.

[12] Kim HA, Lee JK, Kim EK, Seol H, Noh WC (2014). Serum Human Epidermal Growth Factor Receptor 2 Levels as a Real-Time Marker for Tumor Burden in Breast Cancer Patients. Journal of Surgical Oncology.

[13] Carney WP, Neumann R, Lipton A, Leitzel K, Ali S, Price CP (2004). Monitoring the Circulating Levels of the HER2/neu Oncoprotein in Breast Cancer. Clinical Breast Cancer.

[14] Lee SB, Lee JW, Yu JH, Ko BS, Kim HJ, Son BH, Gong G, Lee HJ, Kim SB, Jung KH, Ahn JH, Lee W, Sung J, Ahn SH (2014). Preoperative serum HER2 extracellular domain levels in primary invasive breast cancer. BMC Cancer 14:929.

[15] Schippinger W, Regitnig P, Bauernhofer T, Ploner F, Hofmann G, Krippl P, Wehrschütz M, Lax S, Carney W, Neumann R, Wernecke KD, Samonigg H (2004). The course of serum HER-2/neu levels as an independent prognostic factor for survival in metastatic breast cancer. Oncology reports.

[16] Jaggupilli A, Elkord E (2012). Significance of CD44 and CD24 as cancer stem cell markers: an enduring ambiguity. Clin Dev Immunol.

[17] Kim JS, Kim HA, Seong MK, Seol H, Oh JS, Kim EK, Chang JW, Hwang SG, Noh WC (2016). STAT3-survivin signaling mediates a poor response to radiotherapy in HER2-positive breast cancers. Oncotarget.

[18] Feinberg AP, Ohlsson R, Henikoff S (2006). The epigenetic progenitor origin of human cancer. Nat Rev Genet.

[19] Balic M, Schwarzenbacher D, Stanzer S, Heitzer E, Auer M, Geigl JB, Cote RJ, Datar RH, Dandachi N (2013). Genetic and epigenetic analysis of putative breast cancer stem cell models. BMC Cancer.

[20] Brooks MD, Burness ML, Wicha MS (2015). Therapeutic Implications of Cellular Heterogeneity and Plasticity in Breast Cancer. Cell Stem Cell.

[21] Kyndi M, Sorensen FB, Knudsen H, Overgaard M, Nielsen HM, Overgaard J, Danish Breast Cancer Cooperative G (2008). Estrogen receptor, progesterone receptor, HER-2, and response to postmastectomy radiotherapy in high-risk breast cancer: the Danish Breast Cancer Cooperative Group. J Clin Oncol.

[22] Liang K, Lu Y, Jin W, Ang KK, Milas L, Fan Z (2003). Sensitization of breast cancer cells to radiation by trastuzumab. Mol Cancer Ther.

[23] Tchou J, Lam L, Li YR, Edwards C, Ky B, Zhang H (2015). Monitoring serum HER2 levels in breast cancer patients. Springerplus.

[24] Lian L, Yee M, Fu T, Tchou JC, Zhang H (2012). Challenges in the clinical utility of the serum test for HER2 ECD. Biochim Biophys Acta.

[25] Fehm SB, Duerr-Stoerzerv S, Sotlar K, Mueller V, Wallwiener D, Lane N, Solomayer E, Uhr J (2007). Determination of HER2 status using both serum HER2 level and circulating tumor cells in patiets with recurrent breast cancer whose primary tumor was HER2 negative or of unknown HER2 status. Breast Cancer Research.

[26] Kim JS, Chang JW, Yun HS, Yang KM, Hong EH, Kim DH, Um HD, Lee KH, Lee SJ, Hwang SG (2010). Chloride intracellular channel 1 identified using proteomic analysis plays an important role in the radiosensitivity of HEp-2 cells via reactive oxygen species production. Proteomics.

[27] Kim JS, Kim EJ, Oh JS, Park IC, Hwang SG (2013). CIP2A modulates cell-cycle progression in human cancer cells by regulating the stability and activity of Plk1. Cancer Res.

[28] Wolff AC, Hammond ME, Schwartz JN, Hagerty KL, Allred DC, Cote RJ, Dowsett M, Fitzgibbons PL, Hanna WM, Langer A, McShane LM, Paik S, Pegram MD (2007). American Society of Clinical Oncology/College of American Pathologists guideline recommendations for human epidermal growth factor receptor 2 testing in breast cancer. J Clin Oncol.

[29] Eisenhauer EA, Therasse P, Bogaerts J, Schwartz LH, Sargent D, Ford R, Dancey J, Arbuck S, Gwyther S, Mooney M, Rubinstein L, Shankar L, Dodd L (2009). New response evaluation criteria in solid tumours: revised RECIST guideline. Eur J Cancer.

